# Measuring Relative-Story Displacement and Local Inclination Angle Using Multiple Position-Sensitive Detectors

**DOI:** 10.3390/s101109687

**Published:** 2010-11-01

**Authors:** Iwao Matsuya, Ryuta Katamura, Maya Sato, Miroku Iba, Hideaki Kondo, Kiyoshi Kanekawa, Motoichi Takahashi, Tomohiko Hatada, Yoshihiro Nitta, Takashi Tanii, Shuichi Shoji, Akira Nishitani, Iwao Ohdomari

**Affiliations:** 1 Faculty of Science and Engineering, Waseda University, Okubo 3-4-1, Shinjuku, Tokyo, Japan; E-Mails: satomaya@tanii.nano.waseda.ac.jp (M.S.); iba@tanii.nano.waseda.ac.jp (M.I.); kondo@tanii.nano.waseda.ac.jp (H.K.); kanekawa@kurenai.waseda.jp (K.K.); tanii@waseda.jp (T.T.);shojis@waseda.jp (S.S.);anix@waseda.jp (A.N.);ohdom@waseda.jp (I.O.); 2 Kajima Technical Research Institute, Kajima Corporation, Tamagawa1-36-1, Chofu, Tokyo, Japan; E-Mails: katamura@kajima.com (R.K.); takahamo@kajima.com (M.T.); hatada@kajima.com (T.H.); 3 Department of Architecture, Ashikaga Institute of Technology, Ohmae-Cho 268-1, Ashikaga, Tochigi, Japan; E-Mail: ynitta@ashitech.ac.jp (Y.N.)

**Keywords:** structural health monitoring, local inclination angle, relative-story displacement, position-sensitive detector, PSD

## Abstract

We propose a novel sensor system for monitoring the structural health of a building. The system optically measures the relative-story displacement during earthquakes for detecting any deformations of building elements. The sensor unit is composed of three position sensitive detectors (PSDs) and lenses capable of measuring the relative-story displacement precisely, even if the PSD unit was inclined in response to the seismic vibration. For verification, laboratory tests were carried out using an *Xθ*-stage and a shaking table. The static experiment verified that the sensor could measure the local inclination angle as well as the lateral displacement. The dynamic experiment revealed that the accuracy of the sensor was 150 μm in the relative-displacement measurement and 100 μrad in the inclination angle measurement. These results indicate that the proposed sensor system has sufficient accuracy for the measurement of relative-story displacement in response to the seismic vibration.

## 1. Introduction

Recently, relative-story displacement measurements have attracted much attention because of their capability of directly monitoring building damage [1,2]. Since deformation of building elements results in residual displacement, the displacement measurement is essential for the detection of damage. In particular, measuring the relative-story displacement during earthquakes is effective for real-time structural diagnosis. However, it is difficult to precisely measure the relative-story displacement because the sensor unit is inclined in response to the seismic vibration [3].

A possible solution is to measure the local inclination angle and the torsion angle as well as the relative-story displacement. Such a measurement has already been realized in the image stabilizer of a digital camera by which the captured image is automatically stabilized against unstable handling using an embedded gyro sensor [4]. However, gyro sensors do not work in case of seismic vibrations because the frequency of the seismic motion is too low (from DC to 20 Hz), and the inclination angle (approximately 0.001 rad) is also too small. Park and his group have proposed a method called the partitioning approach that measures the relative-story displacement and the inclination angle of the floor by implementing two video cameras on every floor [5]. However, the video camera approach requires additional computational image processing such as pixel scanning, object identification, and contour definition, and is not suitable for practical use. Moreover, in the partitioning approach, the location where the camera is set up is limited to the open ceiling space in order to simultaneously monitor the motion of targets on the two adjacent upper layers. Thus, the conventional methods are not convenient for the relative-story displacement measurement.

In this paper, we propose a novel sensor system composed of three pairs of position sensitive detectors (PSDs) and light emitting diode (LED) arrays. The three LED arrays are fixed on the ceiling whereas the three PSDs are installed in one place on the floor so that each PSD captures the motion of the corresponding LED array. First, we show that, using this sensor system, the relative-story displacement, the local inclination angle, and the torsion angle can be measured independently. Because the LED light propagates otropically, the inclination angle of the LED, *i.e.*, the bending of the upper layer, is negligible [3]. Next, we evaluate the performance of the sensor system by dynamically moving the PSD unit and the LED array using an *Xθ*-stage and a shaking table. Finally, we discuss the feasibility of this sensor system for monitoring the structural health of a building from the viewpoint of response speed and resolution.

## 2. Sensor Design

**Figure 1.**
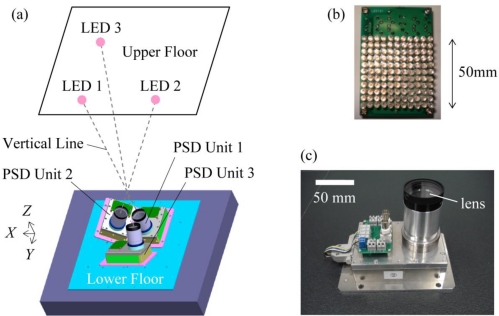
Schematic diagram of the relative-story displacement sensor. **(a)**Arrangement of three LEDs and three PSD units (Birds eye view); **(b)** Photographs of the LED array; and **(c)** the PSD unit.

**Figure 2.**
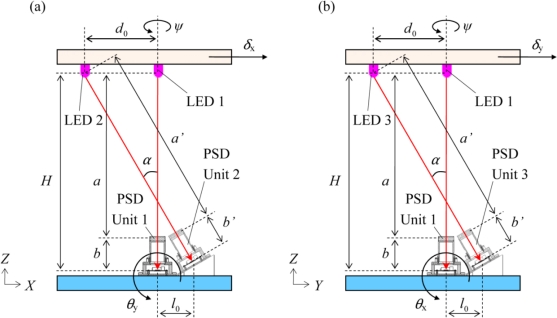
Cross-section view of the relative-story displacement sensor. **(a)** *XZ*-plane view and **(b)** *YZ*-plane view.

Figure 1 shows the schematic diagram of the sensor system, and Figure 2 shows the cross-section view. The specifications of the single PSD unit were reported elsewhere [6,7]. As shown in Figure 1 and Figure 2, the PSD unit1 is located in the original position (0, 0, 0). The PSD unit2 and the PSD unit3 are located at (*l*_0_, 0, 0) and (0, *l*_0_, 0), respectively.

The position of three LED light sources fixed on the ceiling is located at (0, 0, *H*), (–*d*_0_, 0, *H*), (0, −*d*_0_, *H*), respectively. The PSD unit2 is installed with an inclination angle *α* so that the PSD unit2 faces the LED2. The PSD unit3 is also inclined with the angle *α*. Moreover, the three PSD units are rigidly connected with each other and immobilized on the floor. In accordance with the arrangement of the PSD unit1 and the LED1, the distance between the ceiling and the floor is expressed as:

(1) a+b=H

(2) $$\frac{1}{a}+\frac{1}{b}=\frac{1}{f}$$

(3) $$H=\frac{{\left[\left(b/a\right)+1\right]}^{2}}{\left(b/a\right)}\cdot f$$

where *a* is the distance from the lens to the light source, *b* is the distance from the PSD1 to the lens, and *f* is the fixed focal length of the lens. Equation (2) obeys the Gaussian lens formula, and Equation (3) is derived from Equation (1) and Equation (2). In Equation (3), the ratio *b*/*a* represents the magnification of the PSD unit1. In this arrangement, the position of the light spot focused on the PSD1 is expressed as:

(4) $$X_{1}=\frac{b}{a}\left(-\delta_{x}+H\theta_{y}\right)$$

(5) $$Y_{1}=\frac{b}{a}\left(-\delta_{y}-H\theta_{x}\right)$$

where (*δ*_x_, *δ*_y_) is the lateral displacement of the LED1, and (*θ*_x_, *θ*_y_) is the inclination angle of the PSD unit1 [8]. Similarly, the position of the light spot (*X*_2_, *Y*_2_) focused on the PSD2 is expressed as:

(6) $$a\prime +b\prime =\frac{H}{\text{cos }\alpha }$$

(7) $$X_{2}=\frac{b\prime }{a\prime }\left[-\text{cos }\alpha \delta_{x}+(\frac{H}{\text{cos }\alpha }-l_{0}\text{ sin }\alpha )\theta_{y}\right]$$

(8) $$Y_{2}=\frac{b\prime }{a\prime }\left(-\delta_{y}-H\theta_{x}\right)-l_{0}\psi$$

where *ψ* is the torsion angle of the ceiling around *Z*-axis, as shown in Figure 2, and the ratio *b*’/*a*’ is the magnification of the PSD unit2. Since the PSD unit2 is inclined with respect to the vertical line, the distance from the PSD2 to the LED2 differs from the story height *H*, and also the magnification of the PSD unit2 *b*’/*a*’ differs from that of the PSD unit1 (*b*/*a*). For the PSD unit3, we can write:

(9) $$X_{3}=\frac{b\prime }{a\prime }\left(-\delta_{x}+H\theta_{y}\right)+l_{0}\psi$$

(10) $$Y_{3}=\frac{b\prime }{a\prime }\left[-\text{cos }\alpha \delta_{y}+(\frac{H}{\text{cos }\alpha }-l_{0}\text{ sin }\alpha )\theta_{x}\right]$$

**Figure 3.**
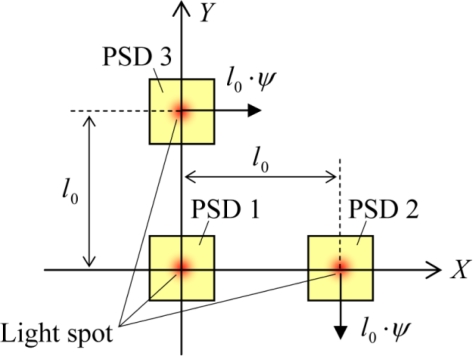
Displacement of the light spot which comes from the LED and is focused on the PSD surface by the lens in response to the torsional motion of the upper layer. The schematic shows the *XY*-plane. The original point for the torsional motion is located at the center of the PSD1. If the upper layer was counterclockwise rotated with the angle *ψ*, the light spot on the PSD3 displaces by *l*_0_·*ψ*in *X*-direction, and the light spot on the PSD2 displaces by *l*_0_·*ψ*in negative *Y*-direction.

We assume that in Equations (4,5,7,10), the inclination angles *θ*_x_, *θ*_y_ and the torsion angle *ψ* are positive when they are rotated counterclockwise, and the center of the rotation is the original point. The movement of (*X*_2_, *Y*_2_) and (*X*_3_, *Y*_3_) in response to the torsional motion of the upper layer is depicted in Figure 3. Here we note that solving the simultaneous equations of (4,5,7,10) is not categorized as a six-degree-of-freedom problem but as a five-degree-of-freedom problem although six variables of (*X*_1_, *Y*_1_, *X*_2_, *Y*_2_, *X*_3_, *Y*_3_) are included. Namely, for solving these simultaneous equations, the Equation (8) need not be taken into account because the variables of (*δ*_x_, *θ*_y_, *ψ*, *δ*_y_, *θ*_x_) can be determined without *Y*_2_ as follows:

(11) $$\left(\begin{array}{c} X_{1}\\ X_{2}\\ X_{3}\\ Y_{1}\\ Y_{3} \end{array}\right)=T\cdot \left(\begin{array}{c} \delta_{x}\\ \theta_{y}\\ \psi \\ \delta_{y}\\ \theta_{x} \end{array}\right)=\left[\begin{array}{ccccc} -\frac{b}{a} & \frac{b}{a}H & 0 & 0 & 0\\ -\frac{b\prime }{a\prime }\text{cos }\alpha & \frac{b\prime }{a\prime }(\frac{H}{\text{cos }\alpha }-l_{0}\text{ sin }\alpha ) & 0 & 0 & 0\\ -\frac{b\prime }{a\prime } & \frac{b\prime }{a\prime }H & l_{0} & 0 & 0\\ 0 & 0 & 0 & -\frac{b}{a} & -\frac{b}{a}H\\ 0 & 0 & 0 & -\frac{b\prime }{a\prime }\text{cos }\alpha & \frac{b\prime }{a\prime }(\frac{H}{\text{cos }\alpha }-l_{0}\text{ sin }\alpha ) \end{array}\right]\left(\begin{array}{c} \delta_{x}\\ \theta_{y}\\ \psi \\ \delta_{y}\\ \theta_{x} \end{array}\right)$$

Equation (11) indicates that (*X*_1_, *X*_2_, *X*_3_) and (*Y*_1_, *Y*_3_) can be calculated independently. Namely, (*X*_1_, *X*_2_, *X*_3_) can be calculated using (*δ*_x_, *θ*_y_, *ψ*), whereas (*Y*_1_, *Y*_3_) can be calculated using (*δ*_y_, *θ*_x_). We note that the relative-story displacement *δ*_x_ depends not on the torsion angle *ψ*, but on the inclination angle *θ*_y_. This indicates that, for measuring the relative-story displacement, we must focus on the inclination angle *θ*_y_ rather than the torsion angle *ψ*, which is sufficiently small in case of seismic building movements. Therefore, we assume *ψ* = 0 in the following experimental setup.

## 3. Experimental Setup

**Figure 4.**
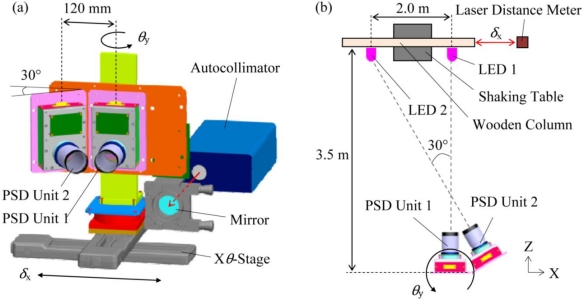
Experimental setup for measuring the relative-story displacement and the inclination angle using two PSD units. **(a)** Front perspective view; and **(b)** *XZ*-plane view.

Figure 4 shows the experimental setup for measuring the lateral displacement *δ*_x_ and the inclination angle *θ*_y_. Two pairs of LED arrays and PSD units were set up laterally at a distance of 3.5 m. To simply verify the methodology, we utilized two PSD units and solved the problem concerning two-degrees of freedom. In the three-pair-combined PSD unit system, the PSD unit2 and the PSD unit3 are arranged symmetrically, indicating that the verification is sufficient by concerning the two-degrees of freedom. As shown in Figure 4, the two pairs of PSD units were immobilized on an *Xθ*-stage with an interval of 120 mm, and the PSD unit2 was connected with the PSD unit1 with the angle of 30°. Two LED arrays were immobilized on a wooden column with an interval of 2.0 m. The wooden column was mounted on a shaking table so that the immobilized LED arrays could move only in the *X*-direction. A laser distance meter was set up nearby the shaking table so as to measure the displacement of the shaking table, and an autocollimator was set up in the back of the combined PSD unit system to measure the inclination angle.

In the static experiment, the shaking table was fixed and the PSD units were displaced using the *X*-stage. The translational movement of ±30 mm in *X*-direction and the rotational movement of ±1.7 mrad in *θ*_y_-direction were measured using the PSD unit1 and the PSD unit2. In this experiment, the values (*δ*_x_, *θ*_y_) measured by the laser distance meter and the autocollimator were used as the reference.

In the dynamic response experiment, the *X*-stage was fixed and the wooden column was vibrated using the shaking table. The inclination angle *θ*_y_ and the displacement *δ*_x_ were simultaneously measured in real time using the PSD unit1 and the PSD unit2 when the shaking table and the *θ*-stage were moved independently. The shaking table was controlled to be vibrated with an amplitude of ±10 mm and a frequency of 0.5 Hz. The *θ*-stage was controlled to be rotated with an inclination angle of ±1.7 mrad (0.1°) and the frequency of 0.9 Hz. The fixed focal length of both the PSD unit1 and the PSD unit2 was 100 mm.

## 4. Results and Discussion

**Figure 5.**
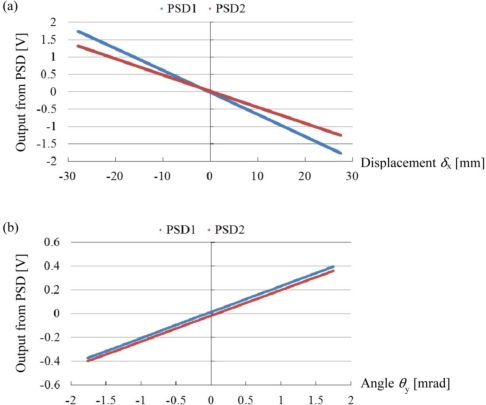
Output voltages from the PSD units according to **(a)** the lateral displacement *δ*_x_ and **(b)** the inclination angle *θ*_y_ in the static experiments.

Figure 5 shows the results of the static experiments. Figure 5(a) shows the output voltages from the PSD unit1 and the PSD unit2 according to the relative displacement of the PSD unit to the light source. In this experiment, only the *X*-stage was displaced, and the *θ*-stage was fixed. Figure 5(b) shows the output voltages from the PSD unit1 and the PSD unit2 according to the rotational movement of the *θ*-stage. In this experiment, the *X*-stage was fixed and only the *θ*-stage was rotated. As shown in Figure 5(a), both two lines exhibit linearity with respect to the displacement of the *X*-stage. Similarly, as shown in Figure 5(b), the two lines exhibit linearity with respect to the rotation of the *θ*-stage. From these results, the following equation is obtained:

(12) $$\left(\begin{array}{l} V_{1}\\ V_{2} \end{array}\right)=\left[\begin{array}{ll} -0.06340 & 218.7\\ -0.04647 & 216.2 \end{array}\right]\left(\begin{array}{l} \delta_{x}\\ \theta_{y} \end{array}\right)$$

where *V*_1_ and *V*_2_ are the output voltages from PSD1 and PSD2, respectively, in the unit of volt, *δ*_x_ is the lateral displacement of the *X*-stage in the unit of millimeter, and *θ*_y_ is the inclination angle of the *θ*-stage in the unit of milliradian. The conversion coefficients from the output voltage to the position of the light spots on PSDs are 0.473 V/mm for the PSD unit1 and 0.484 V/mm for the PSD unit2. Using these conversion coefficients, the Equation (12) can be transformed into the following equation:

(13) $$\left(\begin{array}{l} X_{1}\\ X_{2} \end{array}\right)=\left[\begin{array}{ll} -0.0300 & 103.5\\ -0.0225 & 104.7 \end{array}\right]\left(\begin{array}{l} \delta_{x}\\ \theta_{y} \end{array}\right)$$

Note that (*X*_1_, *X*_2_) is in the units of millimeters, but (*X*_1_, *X*_2_) involves the rotational component in addition to the displacement. The matrix for transforming (*X*_1_, *X*_2_) into (*δ*_x_, *θ*_y_) can be determined as the inverse of the transformation matrix in Equation (13).

We investigated the resolution, which was the most important performance of the combined PSD unit system. The resolution is given by the following equations [9,10]:

(14) $$\left(\begin{array}{l} \Delta X_{1}\\ \Delta X_{2} \end{array}\right)=J\cdot \left(\begin{array}{l} \Delta \delta_{x}\\ \Delta \theta_{y} \end{array}\right)=\left[\begin{array}{ll} \frac{\partial X_{1}}{\partial \delta_{x}} & \frac{\partial X_{1}}{\partial \theta_{y}}\\ \frac{\partial X_{2}}{\partial \delta_{x}} & \frac{\partial X_{2}}{\partial \theta_{y}} \end{array}\right]\left(\begin{array}{l} \Delta \delta_{x}\\ \Delta \theta_{y} \end{array}\right)$$

Equation (14) represents the differential of Equation (13), but the differential matrix *J* in Equation (14) is equivalent to the transformational matrix in Equation (13) since, as shown in Figure 5, all lines are straight lines from the origin, and the differential coefficients do not change. Thus, we can write the differential matrix *J* as follows:

(15) $$J=\left[\begin{array}{ll} -0.0300 & 103.5\\ -0.0225 & 104.7 \end{array}\right]$$

By using the matrix *J*^−1^, the resolution of the combined PSD unit system can be calculated as follows:

(16) $$J^{-1}=\left[\begin{array}{cc} -128.9 & 127.4\\ -0.02770 & 0.03693 \end{array}\right]$$

(17) $$R_{i}=\sqrt{\sum {\left(J^{-1}{}_{\mathrm{ij}}\;\cdot \;\sigma_{j}\right)}^{2}},\;\;(i,j=1,2)$$

where *R*_1_ is the resolution in the displacement measurement, *R*_2_ is that in the inclination angle measurement, and *σ*_1_ and *σ*_2_ are the resolution of the PSDs. We assume that *σ*_1_ = *σ*_2_ = 0.6 μm in accordance with the data sheet from the vendor [11]. From Equations (16) and (17), the resolution for each axis is calculated as follows:

(18) $$R_{1}=\sqrt{{\left(-128.9\times 0.6\right)}^{2}+{\left(-127.4\times 0.6\right)}^{2}}=108.7\;[\mu m]$$

(19) $$R_{2}=\sqrt{{\left(-0.02770\times 0.6\right)}^{2}+{\left(-0.03693\times 0.6\right)}^{2}}=27.68\;[\mu \text{rad}]$$

As shown in Equations (18) and (19), the resolution depends on the angle between two PSD units *α*, the distance between two PSD units *l*_0_, and the focal length of the lens *f*. Thus, we determined these parameters carefully. We determined the distance *l*_0_ (=120 mm) and focal length *f* (=100 mm) in accordance with the dimensions of the PSD unit which was produced previously [5,6]. The angle *α* must be small so as to save the installation space, but the angle *α* must be sufficiently wide so as to enhance the resolution. We conjectured that a resolution of approximately 0.1 mm is required for the structural health monitoring. To achieve such high resolution, the angle between the two PSD units must be wider than 30°. Thus, we determined the angle *α* to be 30°.

**Figure 6.**
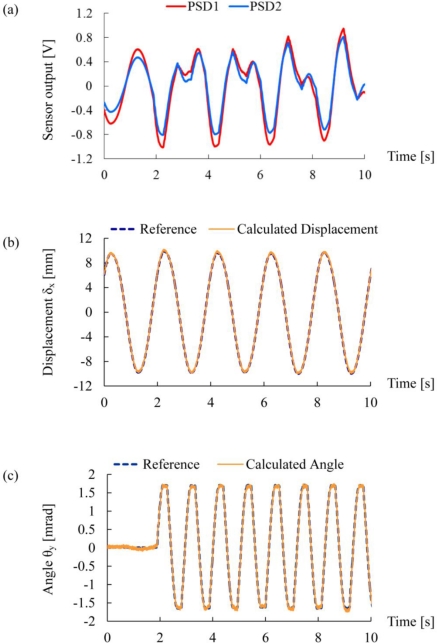
The results of the dynamic experiments using the shaking table and the *θ*-stage. **(a)** The output voltages from PSDs; **(b)** the calculated displacement *δ*_x_; and **(c)** the calculated angle *θ*_y_.

Figure 6 shows the results of the dynamic experiment. In this experiment, the inclination angle *θ*_y_ and the displacement *δ*_x_ were simultaneously measured in real time using the PSD unit1 and the PSD unit2 when the shaking table and the *θ*-stage were moved independently. Thus, the outputs from the PSD unit1 and the PSD unit2 exhibited the mixed motion, as shown in Figure 6(a). The displacement and the inclination angle were calculated using simultaneous Equations (12), and the calculated displacement and inclination angle were depicted in Figure 6(b) and Figure 6(c), respectively. The displacement and angle measured using the combined PSD unit system coincided with the references, as shown in Figure 6(b) and Figure 6(c). The error between the displacement measured by the combined PSD unit system and that measured by the laser distance meter was evaluated to be on average 0.15 mm. The error between the inclination angle measured by the combined PSD unit system and that measured by the autocollimator was evaluated to be 0.1 mrad in average. The values are in good agreement with the resolution of the combined PSD unit system estimated in Equations (18) and (19), indicating that our theoretical model is valid. The results show that the proposed method can correctly measure the relative-story displacement and the inclination angle of the floor.

## 5. Conclusions

A novel sensor system to measure both the relative-story displacement and the local inclination angle was developed using three pairs of PSD units. We established the theory for calculating the relative-story displacement and the local inclination angle from the output voltage of the PSD units and verified the theory by both static experiment and dynamic experiments. The accuracy of the LDS system was experimentally evaluated to be approximately 150 μm in the relative displacement measurement and 100 μrad in the inclination angle measurement. It is clear that the proposed sensor system can measure the relative-story displacement even if the PSD unit is inclined due to the seismic vibration. This indicates that the proposed sensor system can be installed in any point of an actual building. Moreover, because the calculation of the relative-story displacement and the inclination angle is a comparatively easy task, we can realize real-time multipoint measurements. In short, this system is useful for identifying the damage-sensitive elements and evaluating the seismic capacity of the building.
